# Recommendations on treatment for IPF

**DOI:** 10.1186/1465-9921-14-S1-S6

**Published:** 2013-04-16

**Authors:** Jürgen Behr, Luca Richeldi

**Affiliations:** 1 Department of Internal Medicine V, University of Munich, Comprehensive Pneumology Center, Marchioninistr. 15, 81377 Munich, Germany; 2 Centre for Rare Lung Disease, University of Modena and Reggio Emilia, Modena, Italy

**Keywords:** Idiopathic Pulmonary Fibrosis (IPF), recommendations, guidelines, treatment

## Abstract

Patient management in Idiopathic Pulmonary Fibrosis (IPF) is largely based on societal guidelines and recommendations. A recent update by the American Thoracic Society (ATS), European Respiratory Society (ERS), Japanese Respiratory Society (JRS) and Latin American Thoracic Association (ALAT) provided updated guidance on the diagnosis and management of IPF, along with recommendations on pharmacologic and non-pharmacologic approaches to patient management. The treatment guidance is based on GRADE criteria, which rates the quality of evidence according to previously published methodology. Here we discuss how to interpret the recent guideline updates and the implications of this guidance for clinical practice. In addition we discuss the assessment and recommendations for a number of pharmacological agents that have been the focus of clinical trials over the past years. Although no single pharmacological agent was recommended by the guidelines committee, we discuss how since then, more recent data have resulted in the approval of pirfenidone in Europe, and preliminary negative findings regarding the safety of a triple therapy regimen consisting of prednisone, azathioprine and N-acetylcysteine have raised the question of whether it is no longer a treatment option. As clinicians, we must interpret the available guidance and recommendations as we consider each individual patient and as we discuss the available clinical data and the patient’s own preferences in our approach to the management of this disease.

## Introduction

The management of patients with Idiopathic Pulmonary Fibrosis (IPF) is largely based on the recommendations of prominent societies, such as the American Thoracic Society (ATS) and the European Respiratory Society (ERS). The initial recommendations of the ATS/ERS joint committee were made in 2000; however the guidance provided stated that at that time there were no existing data to demonstrate the benefit of any treatment for IPF patients. [1] Since then, a revised, updated recommendations document has been published. The 2011 joint statement of the ATS, ERS, Japanese Respiratory Society (JRS) and Latin American Thoracic Association (ALAT) provided further guidance on the diagnosis of IPF and provided mini-reviews of each of the therapeutic agents that have been used in studies for the treatment of patients with IPF. This document also provided detail of the methodological approach to producing these recommendations. In this paper we discuss the recommendations made by the committee, data that has since become available regarding the use of specific drugs, and also suggest the current implications of these for the IPF physician.

## The treatment recommendations and methodology

The most recent guidelines document provided the overall opinion of a voting committee on questions for each therapeutic agent, for example “*should patients with IPF be treated with corticosteroid monotherapy?*”. The section regarding treatment was structured around these questions. The committee performed a complete systematic review of the literature for the questions focused on treatment. The methodology used for the diagnosis and treatment sections of the 2011 document followed the GRADE system. This previously published [2,3] approach is an evidence-based methodology which rates the quality of available evidence and strength of recommendations, following literature searches and assessment of the available information.

## Interpreting the recommendations

The recommendations made based on the GRADE system are either a ‘strong’ or ‘weak’ recommendation, for or against a specific treatment. The quality of evidence was determined using the ATS GRADE criteria, [3] which identifies all outcomes important to patients and enables differentiation of critical outcomes from those that are important but not critical. Recommendations depend on the available evidence for all patient-important outcomes and the quality of evidence for each. For each treatment considered by the committee, the committee graded the available evidence as either high, moderate, low, or very low, and then made a recommendation for or against the treatment being discussed. Recommendations were based on the majority vote of the committee, and the number of votes for, against, abstinent, and absent were given for each ‘strong’ or ‘weak’ recommendation. The strength of a recommendation reflects the extent to which the treating physician can be confident that the beneficial effects outweigh the undesirable effects of a treatment, for the range of patients for whom the recommendations are intended. [4]

## Implications of these recommendations for IPF clinical practice

While the GRADE recommendations are not widely understood, in our expert opinion, each recommendation has three-dimensional implications. The first is for the patient. If a strong recommendation is made for an IPF treatment, it is likely that most patients with IPF would want to follow this course of action, and only a small number may not. Secondly, and similarly, the implication for the clinician is that most patients should receive the intervention if a strong recommendation is made “for” a specific treatment. The third area of implication is for the policy makers at a given hospital or institution. If a strong recommendation for the use of an intervention is made, this should be adopted as a policy in most situations. Unfortunately, the key message from the guidelines under discussion is that no treatments for patients with IPF are strongly recommended for use (i.e. no treatments received a “strong y”s’ recommendation). The recommendations against most therapies are strong, as there is insufficient evidence supporting their use. However, other treatment recommendations are weak, reflecting the uncertainty of the risks and benefits of the data, and the need for higher quality data. The “weak no” recommendation means that the majority of patients in this situation would not want the intervention, but a minority of patients would want it. In these cases the role of the clinician is of great importance as they need to help the patient make a treatment decision regarding a pharmacological agent with a weak recommendation. Indeed, a weak positive vote should be interpreted as endorsing the use of a particular therapy, even if in only a minority of patients. If a patient is well-informed and strongly wishes to have pharmacological treatment, this should be selected from the agents with “weak no” recommendations based on their individual case. [4] Thus, it could be concluded that the GRADE system is not ideally suited to such a rare condition that has no established current best recommended treatment.

## Guidance on treatment approaches used in practice

The recommendation for corticosteroids in patients with acute exacerbation of IPF is weak; that is, corticosteroids should be used in the majority of patients with acute exacerbation of IPF, but not using corticosteroids may be a reasonable choice in a minority. [4] No randomised, controlled trials have been performed to investigate their use in IPF patients, and long-term steroid use is associated with significant morbidity. [5]

The guidance focuses on reducing morbidity and the places a low value on potential improvements in lung function, due to the low quality of evidence. Treatment with interferon-gamma-1b was also strongly recommended against. Two randomised, placebo-controlled trials of interferon-gamma-1b therapy have been conducted in patients with IPF. In the first, published in 2004, the primary endpoint of improved progression-free survival (PFS) was not met. There was a suggestion of an effect of interferon-gamma-1b on overall survival, although this was not significantly greater than that observed with placebo treatment (p=0.08), and a subgroup analysis suggested an effect on patients with less severe disease at baseline. [6] However, as this was a small study (n=330), another larger trial (INSPIRE Study), investigated the effect on overall survival in a greater number of patients (n=826). Data from this study showed that interferon-gamma-1b did not improve survival for IPF patients. [7] Due to these negative studies, interferon-gamma-1b received the “strong no” recommendation, based on high quality evidence, with a high value placed on treatment cost and potential risks. [4]

We should now discuss those pharmacological agents that received a “weak no” recommendation. N-acetylcysteine (NAC) monotherapy received this recommendation, placing high value on the reduction of treatment-related morbidity and a low value on low quality data. [4] The Idiopathic Pulmonary Fibrosis International Group Exploring N-Acetylcysteine I Annual (IFIGENIA) [8] study investigated triple combination therapy with high-dose NAC (600 mg TID) versus placebo on a “standard” background therapy of prednisone and azathioprine in all IPF patients enrolled. The primary endpoint of the study was change in vital capacity (VC) and diffusing capacity (DLco), and NAC was found to significantly reduce decline in VC and DLco after one year. However, there were numerous limitations to interpreting these data, such as not all patients being included in the intention-to-treat (ITT) analysis, which violated the ITT population paradigm and the omission of a “placebo-only” treatment arm to allow direct comparison versus a negative control. In addition, use of the last observation carried forward (LOCF) method of analysis, which inflates Type I errors and may overestimate treatment effect, and the fact that it was not clear how important the observed effect was with regard to patient relevant outcomes like quality of life, dyspnea, and survival. It is as a consequence of these limitations that the evidence level is considered low quality. In addition, the recent publication of the full results of the Prednisone, Azathioprine, and N-Acetylcysteine: A Study That Evaluates Response in Idiopathic Pulmonary Fibrosis (PANTHER-IPF) study demonstrated that there was an increased risk of death and hospitalizations in patients with IPF treated with a combination of prednisone, azathioprine, and NAC, as compared with placebo. [9] However, desite the suggestion from these conflicting data and recommendations that high dose steroid therapy and azathioprine should not be used in IPF, combined NAC with immunosuppression together has been widely used as a conventional approach to the treatment of IPF. Thus, the recommendation for NAC combination treatment has been heavily debated, but is generally perceived to be an appropriate treatment for patients who are willing to accept possible adverse consequences, even if the expected benefits are small, i.e. a “weak no” recommendation. Although the use of anti-oxidant monotherapy is not necessarily clear from PANTHER, the NAC-monotherapy and placebo arms of the PANTHER-IPF study are continuing.

Anticoagulant therapy also received a “weak no” recommendation from the ATS/ERS/JRS/ALAT committee. Use of anticoagulants has been investigated in a small, unblinded, randomised study in Japan. [10] Anticoagulation plus corticosteroids or corticosteroids alone was administered to patients. A survival benefit was demonstrated in patients treated with anticoagulation therapy, and thought to be due to reduced mortality during hospitalisation for acute exacerbation or respiratory worsening. However, there were significant limitations to this study including the absence of blinding, imbalanced dropout rates, failure to exclude pulmonary embolism as the cause of deterioration and insufficient documentation regarding anticoagulant administration during outpatient treatment. In addition, the ACE-IPF study of coumadin in IPF ended in 2011 due to ineffectiveness and was also associated with an increased risk of mortality in an IPF population who lacked other indications for anticoagulation. [11] This suggests that there is no role for warfarin-type anticoagulants in the treatment of IPF. These data do not exclude, however, that anticoagulant therapy with unfractioned heparin or low-molecular-weight heparin may have beneficial effects in patients with acute exacerbation of IPF, which should be investigated further. [11]

The final agent that received a “weak no” recommendation that was discussed is pirfenidone. Four randomised, placebo-controlled clinical trials have evaluated the treatment of IPF patients with pirfenidone to date. The Phase II study by Azuma et al., was stopped early due to the incidence of acute exacerbations in patients treated with placebo, while none occurred with pirfenidone treatment. [12] A Japanese Phase III study demonstrated a significant effect on decline of FVC and on progression-free survival in patients treated with pirfenidone, compared with placebo. A limitation of this study was that the primary endpoint was changed before unblinding. [13] The multinational CAPACITY studies investigated pirfenidone treatment in IPF patients. The CAPACITY 004 study demonstrated a significantly reduced decline in % predicted FVC compared with placebo. However, the primary endpoint was not met in the CAPACITY 006 study, but pooled data from both studies support the treatment effect of pirfenidone. [14] The guideline committee gave a ‘weak no’ recommendation, with high value placed on costs and side effects and low value on the possible small reduction in pulmonary decline. Again, this treatment may be appropriate in patients who are willing to accept possible adverse effects even if the benefits are small. [4] The majority of committee member abstained (16/31) from voting on pirfenidone, because most were involved in the CAPACITY Trials. In addition, the guidelines were devised before the CAPACITY study data had been published, and without taking into consideration the positive findings of the Cochrane meta-analysis of pirfenidone data. [15] While the FDA refused approval of pirfenidone based on the two CAPACITY Studies, the European Medicines Agency (EMA) approved pirfenidone in Europe in 2011, based on all of the available data, including the Japanese studies and the Cochrane meta-analysis. The discrepancies between the decisions of the FDA, the EMA and the guideline committee demonstrate that there are different ways to interpret these clinical trials. As the quality of evidence at the time of writing the guidelines was considered “low-to-moderate”, this suggests that the complete dataset available to date is likely to have an important impact on the committee’s confidence in the treatment effect of pirfenidone, and this may change their recommendation at some point.

In terms of non-pharmacological treatment, treatment options receiving “strong yes” recommendations were long-term oxygen treatment and lung transplantation. “Weak yes” recommendations were given for pulmonary rehabilitation, corticosteroids in acute exacerbations and for treatments of asymptomatic gastrointestinal reflux disease. There were “weak no” recommendations for specific treatment of pulmonary hypertension in the IPF setting and for mechanical ventilation for respiratory failure due to IPF. [4]

## Conclusions

As discussed, no single pharmacological agent was recommended for use in the treatment of patients with IPF. Currently, the only approved treatment option that we have in Europe is pirfenidone. We, as physicians, must discuss the available options with individual patients and then make a treatment decision with the patient based on their specific values and preferences. As we know, fully informed patients are best placed to make decisions that are consistent with the best available evidence. Since finalising the 2011 IPF guideline document at the end of the year 2010, more data has become available regarding the efficacy and safety of pirfenidone, and also in terms of the safety of immunosuppression-based triple therapy. In addition, some studies have failed with other putative agents, and it is possible to make recommendations on some agents that were considered ‘investigative’ in the 2011 document but have now proven to be ineffective and are no longer an option. Although the 2011 document is useful in many aspects, we feel that an update of the guidelines is essential, as new information from the molecular level to clinical trials have already provided useful information that can help in determining the best way to treat our patients with IPF.

## Competing interest

Dr. Richeldi reports receiving consulting fees from Boehringer Ingelheim, Intermune, Celgene and Gilead, along with lecture fees from Intermune.

J. Behr has received fees for speaking from Actelion, Bayer-Schering, Boehringer-Ingelheim, Encysive, GSK, Pfizer, Lilly, Nycomed, InterMune, Novartis, MSD and has served as consultant/advisor for Actelion, Bayer-Schering-Pharma, Lilly, Pari-Pharma, GSK, Pfizer, Optima, Gilead. J. Behr has also received research grants from Actelion, Bayer-Schering, InterMune, Pari-Pharma and attending international and national congresses sponsored by Actelion, Boehringer-Ingelheim, AstraZeneca, Bayer.

## Acknowledgements

The authors thank C. Trenam, I. Mandic and M. Smith of IntraMed Communications for editorial assistance in the preparation of the manuscript. Development of this article was supported by InterMune AG. 

## Declarations

This article has been published as part of *Respiratory Research* Volume 14 Supplement 1, 2013:IPF in 2011 – Key updates on guidelines and therapeutics. The full contents of the supplement are available online at http://respiratory-research.com/supplements/14/S1. Publication of this supplement was supported by IntraMed Communications with funding from InterMune, AG. InterMune is the manufacturer of pirfenidone, a product mentioned in this article. The supplement originates from presentations given at the “AIR Event: Advancing IPF Research. Working together to translate IPF research into practice” held in Berlin in November 2011. The publication was proposed by IntraMed Communications and developed in consultation with the journal. All articles in the supplement have undergone the journal’s standard peer review process.
